# Response to geometrical visual illusions in non-human animals: a meta-analysis

**DOI:** 10.1098/rspb.2024.0414

**Published:** 2024-06-19

**Authors:** Oxána Bánszegi, Marcos Rosetti, Uriel J. Olivares, Péter Szenczi

**Affiliations:** ^1^ Instituto de Investigaciones Biomédicas, Universidad Nacional Autónoma de México, Ciudad de México, Mexico; ^2^ Unidad Psicopatología y Desarrollo, Instituto Nacional de Psiquiatría Ramón de la Fuente Muñiz, Ciudad de México, Mexico; ^3^ Consejo Nacional de Humanidades, Ciencias y Tecnologías, Ciudad de México, Mexico

**Keywords:** geometrical visual illusion, individual differences, motivation, perception, spontaneous testing, training

## Abstract

Visual illusions have been studied in many non-human species, spanning a wide range of biological and methodological variables. While early reviews have proved useful in providing an overview of the field, they have not been accompanied by quantitative analysis to systematically evaluate the contribution of biological and methodological moderators on the proportion of illusory choice. In the current meta-analytical study, we confirm that geometrical visual illusion perception is a general phenomenon among non-human animals. Additionally, we found that studies testing birds report stronger illusion perception compared to other classes, as do those on animals with lateral-positioned eyes compared to animals with forward-facing eyes. In terms of methodological choices, we found a positive correlation between the number of trials during training or testing and the effect sizes, while studies with larger samples report smaller effect sizes. Despite studies that trained animals with artificial stimuli showing larger effect sizes compared with those using spontaneous testing with naturalistic stimuli, like food, we found more recent studies prefer spontaneous choice over training. We discuss the challenges and bottlenecks in this area of study, which, if addressed, could lead to more successful advances in the future.

## 1. Introduction

There has been long-standing theoretical and practical interest in how animals (including humans) see the world, in particular, how they perceive basic elements of objects, e.g. colour, shape, size and motion. The topic has been even more alluring since we know that the perception of the physical world may sometimes be distorted, with inconsistencies appearing between an observer’s visual percept and a stimulus’ physical characteristics. Such distortions are known as visual illusions [1,2] and are present in everyday life where they may be used to exploit our misguided senses, for example, in restaurants or in marketing ploys [3–5]. More recently, these phenomena have gained recognition as inexpensive, non-invasive tools to study the neurobiology of visual perception in several fields of basic and medical research (e.g. [6,7]). Despite an extensive body of literature covering visual illusions dating back to the fifth century BC [8], the mechanisms underlying the perception of different visual illusions are still unknown and therefore continue as the focus of many ongoing studies. Here, we attempt to gain insights into animals’ perception of visual illusions by comparing commonalities and discrepancies in how different taxa respond to such stimuli under distinct methodological conditions, using information amassed by studies produced in the last decades.

Many illusions are believed to be caused by processes that are coded into the neural networks that mediate our perceptions. These ‘algorithms’ help us make fast decisions by filtering information or speeding up information processing. This is not different for non-human animals. Observational studies suggest that susceptibility to visual illusions is present in several species in various taxa [9], while empirical studies in laboratory contexts have proven that many species are susceptible to a number of visual illusions, with some species perceiving them in the same way and others in the opposite way as humans do [9–12]. The within- and between-species variability in the way animals perceive visual illusions is surprising but remains unexplained. How sensory information is captured and processed by the visual system can vary notably among taxa; such disparities may emerge from anatomical differences in the structure or position of the eyes, neural circuits underlying perception, and the ecological requirements resulting from a species’ evolutionary history. Additionally, even the different methodological approaches of how we test the susceptibility to visual illusions in the same species can lead to contrasting results [13,14].

In recent years, some effort was made to summarize the literature on visual illusion perception in animals in order to draw attention to possible flaws and shortcomings and highlight future research opportunities and promising areas. However, these reviews mostly focused only on a single species or animal group [14–17], a single methodological [13] or biological aspect [18] and none of them provided a quantitative analysis. Thus, the main goal of the present study is to offer a systematic overview of the literature in order to identify which of the biological and methodological factors have the strongest influence on the susceptibility to geometrical illusions in a relatively wide range of taxa using a meta-analytic approach. Since a wide range of factors have been hypothesized to impact the direction or strength of the perception of visual illusions, in the present meta-analysis, we selected the most consistently reported moderators detailed below.

As biological moderators, we examined their (i) *taxonomic class*. We also grouped the species into four broad categories according to their (ii) *habitat* as a proxy for the environment they mostly live in and which may affect their visual perception: terrestrial (spend their life on the ground), aquatic (spend their life underwater), arboreal (spend all or some time of their life above ground surface) or aerial (those whose main locomotion is flight). We also grouped them according to their (iii) *main food composition*: herbivore, omnivore or carnivore, since feeding is a daily task animals must complete. Another possible influencing factor was the lateral or forward (iv) *position of the eyes* and the resulting (v) *overlapping* (*binocular) visual field*, which in turn may reflect the connectivity of their neural circuits.

**Figure 1.**
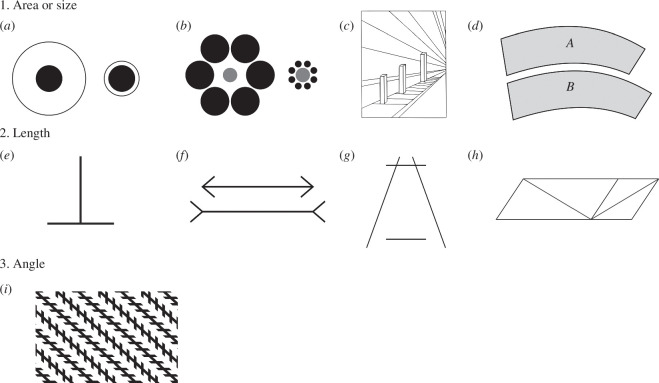
(*a*) Delboeuf illusion: the dot with the smaller surrounding ring appears larger. (*b*) Ebbinghaus illusion: the central circle surrounded by large circles appears smaller. (*c*) Corridor illusion: objects of equal size in a perspective picture appear larger the further away they seem to be. (*d*) Jastrow illusion: one of the identical curved shapes looks larger. (*e*) Vertical–horizontal illusion: observers overestimate the length of a vertical line relative to a horizontal line of the same length. (*f*) Müller–Lyer illusion: of two lines with equal length, the one with the open fins appears longer. (*g*) Ponzo illusion: the upper of two parallel horizontal lines of equal length appears to be longer than the bottom when they are flanked by oblique lines that are closer together at the top than they are at the bottom. (*h*) Sander illusion: the diagonal line bisecting the larger parallelogram appears to be longer than the diagonal line bisecting the smaller parallelogram. (*i*) Zöllner illusion: alternating pattern of crossing lines surrounding parallel lines, creates the illusion that they are not parallel.

As methodological moderators, we first examined the (vi) *type of illusion*. We decided to include only those categorized as geometrical (Corridor, Delboeuf, Ebbinghaus, Horizontal–vertical, Jastrow, Müller−Lyer, Ponzo, Sander and Zöllner; figure 1 for examples of each), which are among the most popular choices for animal testing. Ehm & Wackermann [19] define geometric illusions as those in which the geometrical properties of an object (length, angle, size, etc.) are affected and systematically altered by the presence of other elements in the visual field. Comparing different illusions could reveal interesting information, as we know that humans are more susceptible to some, that susceptibility can change with age and that even seemingly similar illusions may be processed differently by the brain [20–24]. We also further grouped these illusions according to the (vii) *main geometrical property affected*, namely the target object’s area (Delboeuf, Ebbinghaus and Jastrow), length (Horizontal−vertical, Müller−Lyer, Ponzo and Sander) or angle (Zöllner); although some can be treated as exceptions as they can affect area or length depending on how they are presented (e.g. Corridor; figure 1). Next, we classified the studies according to their (viii) *testing method*, which could involve spontaneous choice or training. Testing spontaneous choice involves observing the animals’ natural course of action when presented with the simultaneous alternatives of two or more biologically relevant stimuli (which is also the reward they obtain, e.g. a larger portion of food or easier access to refugia) in an illusory context or without it. In the training approach, individuals undergo practice trials in which neutral stimuli are associated with a reward, which is separate from the stimuli, and their choice according to the training is taken as evidence of the susceptibility to the illusion. While both methods can be used to find the answer to the same question, a recent review by Santacà [13] contrasts the relevance and the potential weaknesses of these methodological approaches while also drawing attention to how they may lead to different results, even in the same species. Since not all the studies involved both sexes, we decided to make an overall analysis of the effect of the (ix) *sex ratio* as well.

We also examined the effect of the (x) *number of training trials*. It has been criticized that usually mammals and birds are subjected to extensive training, sometimes involving thousands of trials, resulting in a much better performance than lower-order vertebrates, such as fishes, which usually are trained with only a few dozen trials [25,26]. Thus, any difference might be associated with the difference in the number of training trials. Similarly, we examined the (xi) *number of testing trials*. In illusion perception studies the desired result is that individual performance should match group performance. However, meeting the significance threshold for the binomial test at the individual level requires the repetition of a larger number of test trials for each subject; thus, increasing the number of trials per individual can result in more individuals reaching the significance threshold for the binomial test, which in turn increases the strength of susceptibility. (xii) *Sample size* was also included as the studies varied considerably among studies.

## 2. Methods

### (a) Search protocol and selection criteria

Where possible, we used the PRISMA statement and checklist guidelines during the preparation of our report [27,28]. We used keyword searches in two online databases (Web of Science and Scopus) on 17 January 2024. We searched for studies containing the following search terms: ‘visual illusion*’ and ‘animal*’. We searched across all years in both databases. We used the ‘Topic’ search field in Web of Science in ‘All Databases’ and the ‘Article title, Abstract, Keywords’ search field in Scopus. This resulted in 984 articles in Web of Science and 329 articles in Scopus. We also collected all papers citing the two influential reviews [9,10] in Web of Science, Scopus and Google Scholar. This resulted in 239 citing articles. Additionally, we extracted the empirical examples cited in these two reviews, resulting in 44 additional articles.

Four additional papers that were not identified by the initial search were accessed because they were cited in the relevant papers. Altogether, the search procedure yielded a total of 1600 papers, and this was reduced to 1220 after removing duplicates. Titles and abstracts of the remaining articles were then screened using the strict inclusion criteria described below.

### (b) Inclusion criteria

A study had to meet several criteria for it to be included in our analysis. First, subjects should be non-human animals. We included only studies that tested geometrical visual illusions (see explanation in the Introduction). Also, we focused on studies that tested adult animals since studies on humans suggest that the emergence of susceptibility to some illusions changes across the life span [29–32]. In addition, only a few studies have been done on young animals [33–38]. We also limited the analysis to experimental studies published in the English language.

The selection and exclusion process of all articles yielded in the search was done by two authors (O.B. and U.J.O.). Six hundred of all 1220 titles and, if necessary, abstracts were judged independently by two authors (O.B. and P.S.). Agreement on inclusion or exclusion of articles between these two researchers was evaluated for consistency, resulting in a Fleiss’ Kappa of 0.901.

**Figure 2.**
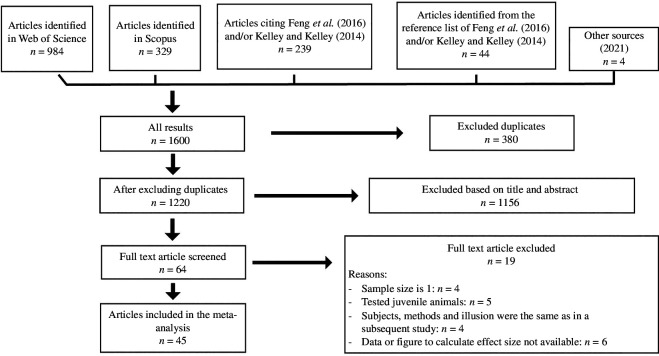
PRISMA flowchart showing systematic search process and study selection.

The full text of 64 articles was accessed and screened and a further 19 were excluded from the analysis yielding a total of 45 papers included in the final analysis (electronic supplementary material, table S1; figure 2). The reasons for exclusion were that (i) the sample size was one (*n* = 4), (ii) researchers tested only juvenile animals (*n* = 5), (iii) the same individuals were tested with the same method as in another article (*n* = 4), and (iv) data and/or figures were not presented in a way which allowed us to extract the necessary information (*n* = 6).

### (c) Calculating effect sizes

To quantify the susceptibility of different illusions in each paper for each of the included species, we needed to extract a standardized effect size from the reported results. First, we extracted the mean proportions of illusory choice either from the data reported, or the plots using WebPlotDigitizer (https://automeris.io/WebPlotDigitizer/) or from personal communication with the authors (electronic supplementary material, table S1). Using the sample size, mean and standard deviation, we also calculated standard errors. We also calculated the effect size for every study as Cohen’s *h* statistic, which is appropriate for proportion values [39]. The confidence interval for the effect sizes was calculated as 95%CI = ES – (1:96*s.e.) to ES + (1:96*s.e.), where ES stands for effect size and s.e. is the asymptotic standard error for the effect size, as suggested by Nakagawa & Cuthill [40]. For comparisons, the absolute value of Cohen’s *h* was used to express the magnitude rather than the direction in which the illusion is perceived.

In 9 out of the 45 studies, we obtained more than one effect size since they tested multiple species with the same illusion (*n* = 5) or tested the same species or individuals with different illusions (*n* = 4). In three cases, papers tested the same individuals with a slightly modified version of the same illusion; there, we used the results only of the first experiment to avoid pseudo-replication by taking into account each individual only once.

### (d) Publication bias

We also examined the final dataset for evidence of bias against publishing studies with small effect sizes or those with small sample sizes; we tested whether there is a relationship between effect sizes or sample sizes and the year the study was published. We also tested whether there is a trend in the use of the two main types of testing methodology: training or spontaneous.

### (e) Statistical analysis

We report the mean proportions of illusory choice and s.e., the magnitude of the illusory choice and s.e. as a forest plot made with the *escalc* and *forest* functions from the packages meta [41] and metafor [42], and Cohen’s *h* with 95% confidence intervals. Using the package ggplot2 [43], we visualized the mean proportions of illusory choice per each of the grouping variables as a combination of violin plots and scatter plots to illustrate the density estimates as well as that of each individual study. Funnel plots were obtained with the *funnel* function.

We executed individual multivariate linear mixed effect models for each individual moderator (as many combinations were missing or unbalanced, e.g. no fish were tested on ‘angle’ type illusions) using the *rma.mv* function [42] and stipulated the study’s ID and the organism species as a random factor. We performed *post hoc* evaluations with general linear hypothesis tests using the *ghlt* function from the multicomp package [44], with Holm’s correction method for multiple comparisons.

Correlations were performed with the *cor.test* function and we opted for the Spearman method, as not all values compared were continuous (e.g. sample size and year of publication). We report the correlation value and confidence intervals. All analysis and graphics were done using R v. 4.1.1 [45]. Significance was set at *p* < 0.05, and all tests were two-tailed.

## 3. Results

### (a) Final dataset and overall phenomena

**Figure 3.**
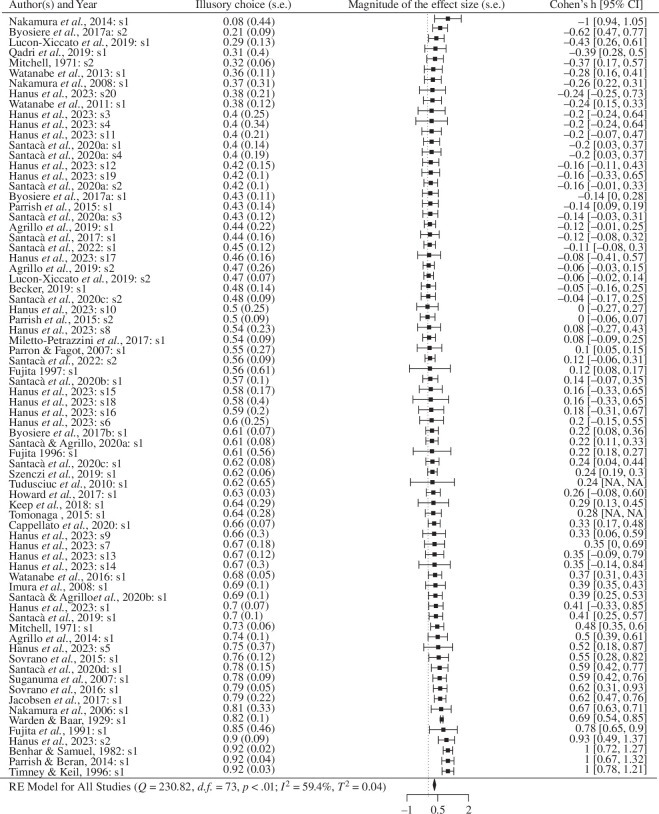
Studies included in the meta-analysis. First column shows the studies included in the meta-analysis. They are arranged from lowest to highest according to the effect size (Cohen’s *h*) of the magnitude of illusion perception. Each line includes the first author’s last name, year and, after colon, experiment number when a paper included more than one species or illusory test (s1, s2 and so on). Other columns, from left to right, show the mean and between parenthesis, the standard error (s.e.) of the proportion of illusory choice, the magnitude (absolute value) of the effect size (Cohen’s *h*) expressed as a forest plot, then the effect size of illusory choice expressed as a Cohen’s *h* 95% confidence interval (CI) between brackets. At the bottom of the plot are the statistical details for the random effect models as well as the summarized effect size for all studies, marked by a black diamond.

The final dataset consisted of 45 studies and 74 effect sizes, testing 534 individuals in total. This included data for 26 species across a broad taxonomic range, tested on 9 different illusions. Figure 3 shows a summary of all studies included. A model for all studies suggests that 59.4% of the observed variance comes from real differences between studies, with a very small standard deviation of the distribution of true effect sizes (*Q* = 230.82, d.f. = 73, *p* < 0.01; *I*^2^ = 59.4%, *τ*^2^ = 0.04). Additionally, the mean magnitude of the effect size of illusory choice, marked by the black diamond at the bottom of figure 3, is in the range of small to medium (0.327).

There was also a statistical difference in the strength of the perception of geometrical illusions depending on whether it reflected the same or opposite way how humans perceive the illusion. Out of the 74 experiments reported here, 31 found no significant effect, 32 found an effect in the same way humans do and only 11 found an effect in the opposite way as humans. Additionally, the magnitude was larger in the same direction with an effect size of 0.477 versus that in the opposite direction, which had an effect size of 0.37 (Est = 0.432 ± 0.12, *p* < 0.001).

### (b) Biological variables

We found statistical differences between the magnitude of the effect size according to the class of the animal tested and their diet composition. The present analysis suggests that birds (0.521 ± 0.27) have a stronger illusory perception than mammals (0.31 ± 0.259), reptiles (0.286 ± 0.22), fishes (0.267 ± 0.184) or the one insect species included in the study (0.26, but significance disappears after adjusting for multiple comparisons), while the other comparisons do not differ from each other. We also found a difference between those animals with lateral (0.376 ± 0.262) and frontally (0.295 ± 0.243) positioned eyes. The evaluation of the relationship between the magnitude of effect size and the binocular visual field also revealed a small negative correlation (−0.237, *p* < 0.04; electronic supplementary material, figure S2*e*). No statistical differences were found between the magnitude of the effect sizes according to the animal’s habitat or main diet composition. The magnitude of the effect sizes for each moderator can be found in the electronic supplementary material, table S2; the statistical values of the *post hoc* comparisons for the moderators can be found in the electronic supplementary material, table S3. Violin plots for each of the biological and methodological factors can be found in the electronic supplementary material, figure S1.

### (c) Methodological variables

When comparing the magnitude of the effect sizes between illusions, we found a significant effect between those with the largest effect sizes, Zöllner (0.478 ± 0.306), Ponzo (0.454 ± 0.353) and Müller–Lyer (0.412 ± 0.195), and with the smallest, Jastrow (0.155 ± 0.116), and between Zöllner and Sander (0.173 ± 0.06). After performing *post hoc* pairwise comparisons and then the corrections for multiple comparisons, however, any significance was lost (see electronic supplementary material, table S3). When grouping the illusions according to the main geometric feature affected, we found ‘angle’ type illusions (0.478 ± 0.306) had bigger effect sizes than ‘length’ type (0.352 ± 0.25) and ‘area’ type (0.281 ± 0.238) illusions; also, ‘length’ had bigger effect than ‘area’ type illusions (see electronic supplementary material, table S3). Although we found no statistical difference between the magnitude of the effect sizes in studies that trained the individuals as compared with those that made spontaneous choice tests, we did find between studies that used artificial (0.397 ± 0.272) or natural stimuli, food (0.268 ± 0.218) and holes (0.116 ± 0.011).

We also evaluated the relationship between the magnitude of effect size and the number of trials during the training methods, number of trials during testing, sex ratio and the sample size. We found moderate to strong correlations between the magnitude of effect size with the number of training trials (0.571, *p* = 0.013) and with the sample size (−0.472, *p* < 0.001) and small correlations with the number of testing trials (0.222, *p* = 0.059), both with control (0.235, *p* = 0.047) and illusion trials (0.264, *p* = 0.025). No correlation was found between the magnitude of effect and sex ratio (0.158, *p* = 0.186). The scatterplots visualizing the correlations can be found in the electronic supplementary material, figure S2.

### (d) Publication bias

A rank correlation test for the funnel plot asymmetry of raw effect size (Kendall’s tau = 0.016, *p* = 0.845), as well as a visual inspection of the funnel plots of the standard error and the inverse standard error, did not suggest publication bias (electronic supplementary material, figure S3). Additionally, we found a positive correlation between the publication year and sample size (*r* = 0.33, *p* = 0.004; electronic supplementary material, figure S4*a*) and a negative correlation between the publication year and magnitude of effect size (*r* = −0.39, *p* < 0.001; electronic supplementary material, figure S4*b*).

When comparing the publication year, we found that older studies tended to train the animals to respond, although the dates span for a large range (mean year 2007 ± 18.3), while more recent studies tend to implement spontaneous testing (mean year 2019 ± 2.15; electronic supplementary material, figure S4*c*).

## 4. Discussion

In general, we found that more than half (43) of the mean proportions of illusory choice differ from chance. Also, geometrical illusions are a perception phenomenon present in at least one case for every class reported in the studies included in the current meta-analysis. Our study also supports the hypothesis that some of the biological and methodological moderators can influence the strength of susceptibility to geometrical visual illusions.

Except for certain taxonomic groups, research instances are limited, sporadic and generally scarce. Only four of the five taxonomic classes of vertebrates and only one taxonomic class of invertebrates are represented in the meta-analysis. Clearly, an area of opportunity for future research is to focus on the underrepresented taxonomical groups in the field of visual illusions; as evidenced here, there are no studies for the vast majority of insects, nor on amphibians and only a few on reptiles. From the classes included in the study, birds appear to be the most susceptible to geometric illusions, although this result should be treated with caution (see below for the relationship between the sample size and the effect size).

A surprising finding is that the position of the eyes seems to influence the strength of perception of visual illusions: animals with forward-facing eyes and large overlapping visual fields (which are mainly represented by primates) have weaker illusory perception than species with laterally positioned eyes (mostly birds and fishes). This result should be interpreted with caution since we cannot determine whether susceptibility to illusions is due to eye placement *per se* or phylogeny. However, a possible explanation for this phenomenon is that animals with laterally positioned eyes scan a large part of the environment with only one eye at a time; thus, they might rely more on contextual information when evaluating the characteristics (e.g. size) of the objects. Animals with mostly stereoscopic vision, however, might get a more punctual information since their visual system gathers information from two different angles. Additionally, the perception of size and illusions seems to be affected whether we perceive it binocularly or monocularly; for example, monocular cues appear to be preferably used for size perception [46], but for instance, the magnitude of the Ponzo illusion is much greater when the Ponzo context is viewed binocularly than monocularly [47].

These findings highlight some of the gaps in the field of visual illusion research. A more systematic evaluation could provide an opportunity to evaluate the effect of class/habitat/visual field permutations by testing, for instance, water-bound mammals (e.g. dolphins [48]; however, only one animal was tested), flightless birds (e.g. ostriches or kiwis) or flying mammals (e.g. bats). Another possibility for future studies is to test mammals that have lateral-positioned eyes, such as most ungulates and rodents or test birds belonging to the order of Strigiformes (i.e. owls). Since ecological niche requirements or even human selection may have shaped the morphological appearance of even closely related species in various directions (e.g. dogs), that also provides a good opportunity when selecting model species.

We found only one study on invertebrates that met the criteria for inclusion in the present analysis [49]. Howard and colleagues [49] showed that bees are also susceptible to Delboeuf illusion, but only if the viewing distance of the animals was not restricted. This and some previous findings on the susceptibility of invertebrates to other types of visual illusions [17,50,51] support the two main hypotheses in the field. First, the mechanisms underlying the perception of illusions (at least some of the geometrical illusions) may be very simple. Second, despite their different eye structures, arthropods share many structural, functional and evolutionary features with vertebrates [52], supporting the idea that insect and vertebrate visual systems share a common evolutionary origin.

The type of illusions seems to significantly influence the magnitude of susceptibility, but after correcting for multiple comparisons, significant values were lost. A better understanding of the perception of each type of illusion could be managed if studies incorporated the susceptibility scores for each individual tested. Recent studies in humans have focused on intra- and inter-subject variability of susceptibility to different visual illusions and show that the magnitude of perception by the same individual can be very different [20,22]. Only a weak connection, if any, has been found between the perception of different types of illusions even when they are considered to belong to the same category [21,24]. This supports the notion that there is no unique common neural path that can account for the perception of all visual illusions. Studies testing the same individuals with different illusions and reporting the relationship between the strength of perception should be favoured. Also, the field would benefit from broadening the range of visual illusions being evaluated: while some are used to test several species (e.g. Delboeuf), others have been barely studied (e.g. Jastrow). Progress in this direction might also change our approach to illusion categorization—while in the current study, we arbitrarily grouped them according to their appearance, a better approach would be to group them in terms of the direction and magnitude in which they can be perceived in an effort to approximate a classification based on the underlying neural mechanisms [1,53–56].

The sample size also significantly influences the strength of the response to an illusory effect at the group level; studies that show a large effect (≥ 0.80) also have remarkably low sample sizes (*n* = 2–5). The negative correlation between the sample sizes and effect sizes suggests that the fewer individuals are tested, the greater the effect found. This happens most likely because individuals that are slower to train or show no interest in the test are often excluded from the studies. We would therefore like to emphasize the importance of proper sample size selection and clarity when reporting the individual data (as well as that of any excluded animals). Larger samples can also help to elucidate individual differences in visual perception, since, for example, the susceptibility to a certain type of visual illusion can vary markedly between individuals.

Regarding the methodological moderators, we found the type of stimuli seems to influence the magnitude of visual illusion perception. In addition, studies using spontaneous choice tasks reported smaller effect sizes than those with trained animals. While this difference was not significant, it suggests that when planning a study, one should consider how the main question of the study influences the methodological choice: training animals might be the better choice if the aim of the study is to reveal the neurological substrate involved in the perception of an illusion as it may lead to a more consistent and stronger effect size with a lower sample size. It is important to note, however, that with training, we cannot exclude the possibility that such processes may involve reinforcement learning towards other environmental cues and not only those responsible for illusory perception (e.g. when training an animal for the Delboeuf illusion, they may respond to the ratio between the plate and the food or the size of the plate on the training trials rather than on the actual illusory factor). If the question involves understanding how the visual illusion is perceived as part of an animal’s natural behavioural repertoire, using spontaneous choice may be the better methodological approach, even if it involves testing a larger number of subjects and may yield lower effect sizes (for additional comparison of the two methods, see [13,26]). Realistically, selecting a testing method freely may not always be possible, as spontaneous testing might not be suitable for all species (e.g. [57] where it was suggested that spontaneous choice test may invoke an inhibitory control problem in dogs). Finally, both methodologies have the issue of evaluating visual acuity. While training may filter out those individuals who consistently perform badly on control tests, in spontaneous choice tests, even with proper controls, it may be impossible to differentiate between visual problems and lack of motivation, which is not enough to interpret null results as a lack of susceptibility. An open challenge is developing visual acuity tests that are valid across taxa.

An ancillary methodological observation is that almost all spontaneous choice tests rely on food as a stimulus. Food is a universal motivator, but not the only thing an animal needs. A clever deviation from food-based stimuli is the study methods of Santacà *et al*. [58], where guppies were tested using a paradigm involving different sizes of holes that the animals needed to cross, then modifying the environment around the holes in order to create the illusory effect. The development of new testing paradigms can also give the opportunity to spontaneously test animals in more naturalistic situations, or even in their natural environment. This could provide information about the acquisition and relevance of certain sensory information in an animal’s everyday life. Finally, testing the susceptibility of the same illusion but in different contexts would also provide the opportunity to cross-validate the phenomena within the same species or reveal that it is limited to a single scenario, providing clues as to which mechanisms may be behind it (i.e. relevant for food but not for mating or escaping).

Some species perceive geometrical visual illusions in the way opposite to that of humans. Although, our analysis shows that the effect is stronger if the perception is the same as in humans. However, this result can be misleading. As there is greater interest in the visual perception of species that are phylogenetically closer to humans, such as mammals, this result may be biased. As shown here, many more mammalian species are chosen as test subjects, while studies in other taxa (e.g. birds and fishes) where species are more likely to perceive illusions in the opposite way that humans do are less numerous. This can be an interesting research line for future studies since, for example, for a while, it seemed birds perceive the ‘area’ type of geometrical visual illusions (i.e. Delboeuf and Ebbinghaus) in the opposite way as humans (see electronic supplementary material, table S1); however, a more recent study with budgerigars found that they perceive the Delboeuf illusion the same way as humans do [59].

We also found a positive correlation between the number of training trials and effect sizes. Some mammals (i.e. primates and dogs) and birds are often subjected to extensive training, which has led to criticism as this may result in better performance than lower-order vertebrates, such as fish, which usually are trained with only a few dozen trials [25,26]. The studies by Bisazza *et al*. [25] using a numerical discrimination task in guppies also support the notion that increasing the number of training trials could result in discrimination choices that could drive an individual’s performance towards significance. Training can also introduce a survival bias, as those who successfully complete the training may be those who are most sensitive to the illusions; Miletto Petrazzini *et al*. [60] showed that those guppies that were faster in learning the numerical rule in the training phase were also the more sensitive to a certain visual illusion. We also found a positive correlation between the number of trials during testing and the effect sizes. As mentioned above, in this field of study, it is often important that the individual performance matches that of the group. Increasing the number of testing trials is one way to achieve this, as it can help to meet the significance threshold for the binomial test at the individual level (cf. [34,61]). However, this can lead to two problems: animals might habituate to the task, get satiated or they can associate additional rules and show a learning effect.

Publication bias was present in the current dataset and showed a trend with time; recent publications show smaller effect sizes and larger sample sizes and opt for spontaneous testing. Fortunately, as is commonly observed in other fields, bias against publishing studies reporting small effects or non-significant results seems to be fading [62]. Also, while training animals used to be the classical approach in illusory perception studies, spontaneous testing seems to be gaining ground as the method of choice. Additionally, we would invite research groups that have established model species to expand their tests to a wider range of illusions, allowing the field to have a better comparability of susceptibility between taxa [63]. Another major gap and, therefore, a future opportunity for the field is the testing of young or senescent non-human animals, as research on these age groups is almost non-existent (and, thus, excluded from the current meta-analysis). From the literature on humans, we know that illusions can differ in their susceptibility by age groups (e.g. [23]); however, this type of research is completely lacking in animals. To our knowledge, only a handful studies were done on young animals [33,35–38] and only one of these has been on mammals [34], which actually found a phenomenon similar to that in humans. Thus, one of our suggestions for future studies is to consider the age of the subjects, not mix immature or ageing animals, but use the opportunity and extend our knowledge by reporting the results of these individuals separately.

Finally, since most modern journals have the option to include supplementary material or have even made sharing raw data via online repositories compulsory, an effort should be made by authors and reviewers to ensure the availability of data at the individual level. In statistical terms, the bare minimum required should be to include the systematic report of the mean, standard deviation and effect sizes. Additional standardization of the number and type of trials during spontaneous food choice tasks should also be beneficial for researchers studying specific species to easily implement the methods and contribute to the pool of information.

## Data Availability

Dataset can be accessed at Figshare [64]. Supplementary material is available online [65].
